# Prevalence and Correlates of Suspected Nonalcoholic Fatty Liver Disease in Chinese Children

**DOI:** 10.3390/ijerph14050465

**Published:** 2017-04-27

**Authors:** Peige Song, Jinyue Yu, Manli Wang, Xinlei Chang, Jiawen Wang, Lin An

**Affiliations:** 1 Department of Child, Adolescent and Women’s Health, School of Public Health, Peking University, Beijing 100191, China; peigesong@hsc.pku.edu.cn (P.S.); 18354226871@163.com (M.W.); changxl_echo@163.com (X.C.); 2 Centre for Population Health Sciences, University of Edinburgh, Edinburgh EH8 9AG, UK; 3 Division of Medicine, School of Life and Medical Science, University College London, London WC1E 6BT, UK; rmhajyy@ucl.ac.uk; 4 Institute of Medical Humanities, Peking University, Beijing 100191, China; 1310120138@pku.edu.cn

**Keywords:** nonalcoholic fatty liver disease, children, prevalence, China

## Abstract

Nonalcoholic fatty liver disease (NAFLD) has become a serious public health problem worldwide; however, the availability of information on the prevalence of NAFLD in the general pediatric population is still limited. The primary aim of this study was to reveal the prevalence and correlates of suspected NAFLD in Chinese children at the national level. Data from the China Health and Nutrition Surveys (CHNS) was used. Weight, height, waist circumference (WC), blood pressure (BP) were measured for children aged 7–18 years. Blood samples were collected and analyzed. Children were classified as having suspected NAFLD if common causes of liver disease were excluded, and serum alanine aminotransferase (ALT) values were above the established thresholds (>22.1 IU/L for girls and >25.8 IU/L for boys). A percentage of 9.03% (75 out of 831) of Chinese children was found to have suspected NAFLD. Overweight and obesity according to BMI percentiles, abdominal obesity, hyperuricemia (uric acid (UA) > 327 μmol/L), and elevated total cholesterol (TC) were all detected as the correlates of childhood suspected NAFLD when adjusting for other factors. Our study revealed the prevalence of suspected NAFLD in general Chinese children at the national level for the first time. Our findings indicate that suspected NAFLD in children is associated with increasing childhood morbidities, further studies are needed to better understand the prevalence of childhood NAFLD and its correlates, and large-scale programs should be launched to screen NAFLD in the pediatric population in China.

## 1. Introduction

Nonalcoholic fatty liver disease (NAFLD) refers to a wide spectrum of hepatic pathology, ranging from a simple liver steatosis (an intrahepatic accumulation of fat) to steatohepatitis [1,2,3]. It could progress to cirrhosis and end-stage liver disease [4,5]. NAFLD is suggested to be the liver presentation of the metabolic syndrome that associates significantly with a cluster of risk factors, namely obesity, insulin resistance, and type 2 diabetes mellitus [4,6,7]. The most important risk factors for NAFLD are the gender of male, increasing age, obesity [5,8,9]. Epidemiological studies show that the prevalence of NAFLD is 80–90% in obese adults, 30–50% in patients with insulin resistance, and approximately 90% in hyperlipidemia patients [6,10].

Over the past decade, NAFLD has rapidly become one of the most common chronic liver diseases worldwide. The prevalence of NAFLD in the general population of developed countries is 20–30% [4]. Evidence suggests that the environmental background for the development of NAFLD may be established in early life where pediatric NAFLD is of primary origin typically [6,10]. Pediatric NAFLD is currently considered to be a global health issue, with approximately 3% of the general pediatric population estimated to be suffering from this disease [10,11]. During the last decade, the prevalence of NAFLD among children has increased approximately 3–5% with a male-to-female ratio of 2:1 [12]. An American national survey indicates that the elevated surrogate marker of NAFLD, which is also known as serum alanine aminotransferase (ALT), is present in 8.0% among the US adolescents ageing from 12 to 19 years [13]. Recent research indicates that the development of NAFLD in children may be strongly associated with obesity [6,14]. Evidence from population-based prevalence studies of pediatric NAFLD suggests that the prevalence of NAFLD ranges from 40 to 70% among obese children [15].

However, since the methods that used to define NAFLD are different, the exact prevalence of NAFLD in children is inconsistent [12]. A better understanding of the epidemiology of pediatric NAFLD and the influence of gender, metabolic and environment factors is essential to provide additional insight into the pathophysiology of NAFLD [16]. In China, the available data suggests an NAFLD prevalence of 5.0% in urban school-aged children and adolescents [17]; however, no other studies have been conducted to reveal the NAFLD prevalence at the national level. In this study, we used data from the China Health and Nutrition Surveys (CHNS) in 2009, a national population-based study, to assess the prevalence of NAFLD in Chinese children and to evaluate its possible correlates [18]. In this study, ALT was chosen as a surrogate for suspected NAFLD in children. To explore the influence of evaluation methods on the NAFLD prevalence, we conducted a comparison with a previous Chinese pediatric NAFLD study, which reported the NAFLD prevalence in urban school-aged children and adolescents using ultrasonography [17]. In addition, to explore the influence of environments (society, economy, behaviour, diet, etc.), we made a comparison with the suspected NAFLD prevalence in Chinese American children using the same definition of suspected NAFLD [19].

## 2. Materials and Methods

The study design and methods of CHNS have been described in detail elsewhere [20]. A multistage random cluster sampling method was adopted in nine provinces (Guangxi, Guizhou, Heilongjiang, Henan, Hubei, Hunan, Jiangsu, Liaoning, and Shandong) with different geographies, economic development levels, and health indicators. As an ongoing national representative household-based study, CHNS has been conducted successively in the years of 1989, 1991, 1993, 1997, 2000, 2004, 2006, 2009, and 2011 [18,20]; however, fasting blood samples were only collected in 2009 from individuals equal to or older than 7 years [20,21], so only the data of CHNS 2009 was adopted because of the availability of biomarker information. At the individual level, the average response rate was 88% for each wave of the survey from 1989 to 2006 [21]. The study protocols were approved by the Institutional Review Boards of the University of North Carolina, Chapel Hill and the Chinese Centre for Disease Control, and all children and their parents provided written informed consent [20].

Demographic data, which included age, gender, residence, was collected using a standardized questionnaire. Anthropometric measuring was conducted by well-trained examiners following standard procedures. Weight was measured to the nearest 0.1 kg with lightweight clothing on a calibrated beam scale. Height was measured to the nearest 0.1 cm without shoes using a portable stadiometer. BMI was calculated as weight in kilograms divided by the square of height in meters. Waist circumference (WC) was measured at a midpoint between the lowest rib and the iliac crest in a horizontal plane using non-elastic tape. Blood pressure (BP) was taken in triplicate after a 10 min seated rest using a mercury sphygmomanometer, an appropriate size of cuff was used, and Korotkoff Phase 1 and Korotkoff Phase 5 were used for defining systolic blood pressure (SBP) and diastolic blood pressure (DBP) [22].

Fasting blood samples were collected from household respondents aged 7 years and older by trained nurses. Then, the blood samples were tested immediately for glucose and hemoglobin A1c. Plasma and serum samples were then frozen and stored at −86 °C. All samples were finally analyzed in a national central lab in the capital with strict quality control. ALT was measured using the International Federation of Clinical Chemistry enzyme method, hemoglobin (Hb) using the volume, conductivity, and light scatter method, serum uric acid (UA) using the enzymatic colorimetric method, total cholesterol (TC) using the cholesterol oxidase phenol aminophenazone method, high-density lipoprotein cholesterol (HDL) and low-density lipoprotein cholesterol (LDL) using the enzymatic method, and triglyceride (TG) using the glycerol-3 phosphate oxidase phenol aminoantipyrine enzymatic method.

ALT levels >22.1 IU/L for girls and >25.8 IU/L for boys were defined as the criteria of suspected NAFLD with no common causes of liver disease during the last four weeks [19]. Overweight was defined as a BMI < 95th percentile and ≥85th percentile for gender and age, obesity was defined as a BMI ≥ 95th percentile (age- and gender-specific) according to the Chinese pediatric BMI classification [23]. Abdominal obesity was defined as a WC ≥ 90th percentile for gender and age according to the Chinese pediatric WC classification [24]. Prehypertension was defined as an SBP and/or a DBP < 95th percentile and ≥90th percentile for age, gender, and height, and hypertension was defined as an SBP and/or a DBP ≥ 95th percentile for age, gender, and height according to the “Fourth Report on Diagnosis, Evaluation, and Treatment of High Blood Pressure in Children and Adolescents” [22,25]. Anaemia was defined according to the World Health Organization criteria as an Hb < 130 g/L for boys aged ≥15 years, <120 g/L for girls aged ≥15 years, <120 g/L for children aged ≥12 and <15 years, and <115 g/L for children aged ≥5 and <12 years [26]. Hyperuricemia was defined using three thresholds of a UA value >327 μmol/L, ≥357 μmol/L, and >416 μmol/L, respectively. There is still no universally accepted threshold for the pediatric population [27]. Dyslipidemia was defined as at least one abnormal value for HDL, LDL, TC, or TG: low HDL was defined as an HDL < 0.9 mmol/L, elevated LDL as an LDL > 3.4 mmol/L, elevated TC as a TC > 5.1 mmol/L, and elevated TG as a TG > 1.7 mmol/L [28].

Descriptive analysis was conducted to describe the prevalence and characteristics of suspected NAFLD, categorical data were expressed as percentages, and continuous data were expressed as means ± standard deviation (SD), for ALT, data were expressed as median with lower and upper quartiles because of its skewed distribution. Comparisons of continuous data and categorical data were performed by *t*-test and χ^2^-test respectively, and by Mann–Whitney U test for ALT. The gender- and age-specific prevalence of suspected NAFLD were calculated. Binary logistic regression models were adopted to examine the association between the presence of suspected NAFLD and its correlates, the association was assessed by the forward selection (likelihood ratio), both univariate and multivariate adjusted odds ratios (ORs) with 95% confidence intervals (CIs) were reported. All the analysis was conducted by SPSS 22.0 (IBM Corporation, Armonk, NY, USA), and a two-tailed *p* value < 0.05 was considered statistically significant.

## 3. Results

A total of 831 children were included, among them, 456 (54.9%) were boys and 375 (45.1%) were girls, 347 (41.8%) children came from urban areas while 466 (56.1%) came from rural areas. Boys had higher WC, Hb, UA, and ALT levels than girls, whereas girls showed higher levels of TC than boys. The included children living in the urban areas were older and had higher levels of weight, height, BMI, and WC, but a lower level of ALT than children in the rural areas. The detailed demographic and clinical characteristics of the children are listed in Table 1.

**Figure 1:**
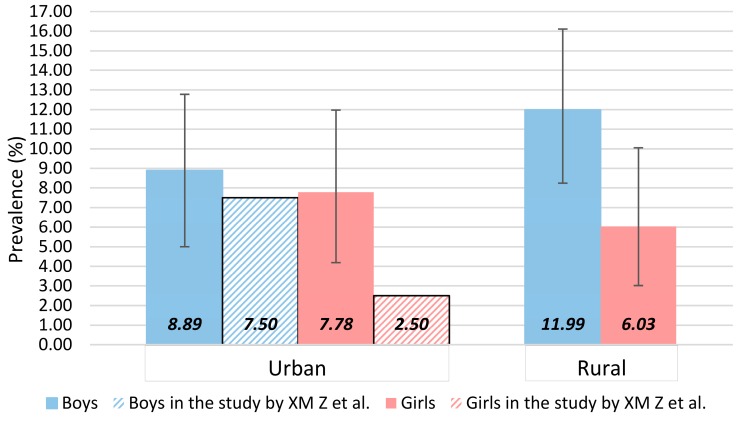
Gender- and residence-specific prevalence of childhood suspected nonalcoholic fatty liver disease (NAFLD) and the comparison with the results from the study by XM Zhang et al. (data from [17]).

A total of 75 children were classified as having suspected NAFLD, among whom 49 were boys and 26 were girls; 29 came from urban areas, while 44 came from rural areas. An overall prevalence of 9.03% (95% CI: 7.22–11.31) was yielded. The prevalence was 10.75% (95% CI: 8.11–13.60) in boys, and 6.93% (95% CI: 4.53–9.60) in girls. In urban children, the prevalence was 8.36% (95% CI: 5.48–11.24), and in rural children, the prevalence was 9.44% (95% CI: 6.87–12.02). The gender- and residence-specific prevalence of childhood suspected NAFLD is shown in Figure 1. The prevalence of suspected NAFLD was higher in our study than that in the study by XM Zhang et al. in both boys and girls [17], especially for urban girls, the prevalence of suspected NAFLD in our study was more than three-fold that in the study by XM Zhang et al. (7.78% vs. 2.50%) [17].

**Figure 2:**
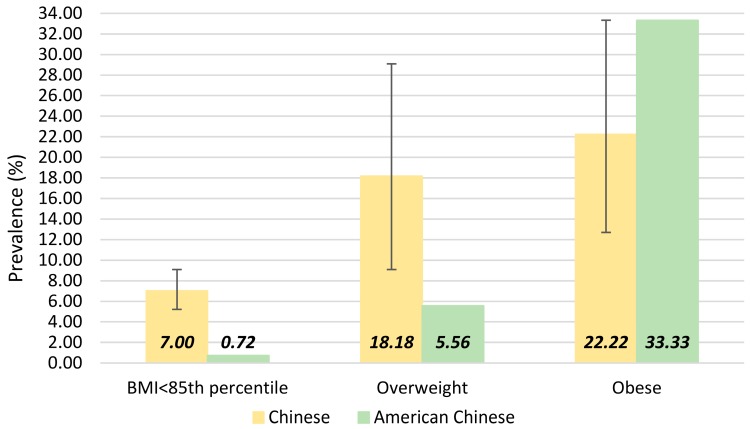
Prevalence of childhood suspected NAFLD according to different BMI categories and the comparison with the results in American Chinese children (data from [19]).

In different BMI categories, the prevalence varied (Figure 2). The prevalence of suspected NAFLD was 7.00% (95% CI: 5.22–9.09) in children with BMI < 85th percentile, 18.18% (95% CI: 9.09–29.09) in overweight children and 22.22% (95% CI: 12.70–33.33) in obese children. The comparison of the prevalence of suspected NAFLD between Chinese and American Chinese is also presented in Figure 2 according to different BMI categories, the estimates of suspected NAFLD in these two populations were both based on the ALT threshold of >22.1 IU/L for girls and >25.8 IU/L for boys [19]. Overall, Chinese children had a higher prevalence of suspected NAFLD than American Chinese children (9.03% vs. 6.14%). The prevalence of suspected NAFLD was much higher in Chinese children with normal weight and overweight children (7.00% vs. 0.72% and 18.18% vs. 5.56%, respectively), whereas the prevalence of suspected NAFLD in obese children was higher in American Chinese than in Chinese (33.33% vs. 22.22%).

The comparison of characteristics of the children with and without suspected NAFLD is shown in Table 2. Children with suspected NAFLD had significantly higher values of weight, BMI, WC, SBP, DBP, UA, ALT, and lipids (TC, LDL and TG), but a lower value of HDL than children without suspected NAFLD.

From the results of binary logistic regression analysis (Table 3), overweight and obesity according to BMI percentiles, abdominal obesity, hyperuricemia (UA > 327 μmol/L) and elevated TC were all correlates of childhood suspected NAFLD.

## 4. Discussion

The importance of screening NAFLD in the pediatric population has been well demonstrated [29,30]. Similar to adults, NAFLD in children also has the potential to progress into cirrhosis and result in significant liver-related morbidity [30]. Although NAFLD is strongly associated with obesity, and several population-based studies only limited the screening of childhood NAFLD in obese children [31], NAFLD has still been demonstrated as an important public health problem in non-obese children and adolescents [31,32]. To the best of our knowledge, the findings of our study provided insights into the prevalence of suspected NAFLD and its correlates in Chinese children for the first time.

At the population level, the NAFLD prevalence is estimated to be between 3% and 10% in children [33], the variation mainly comes from the specific characteristics of the investigated population and diagnostic methods. Although a liver biopsy is the current gold standard for diagnosis [30], its invasive measure is not suitable for the pediatric population for screening purposes. Both blood tests (i.e., elevated ALT) and ultrasound are popular indirect measures of NAFLD in large population studies [16,34]. Comparing with the results of a previous study conducted in urban Chinese children [17], our nation-wide study presented a higher prevalence of suspected NAFLD. There may be two reasons: First, the population composition in these two studies was different; the included children in our study came from both urban and rural areas across the whole country. In our analysis, suspected NAFLD prevalence in the urban areas was lower than in the rural areas. The study by XM Zhang et al. was only conducted in an urban area of Yangtze River delta region [17]. Second, the screening methods were different; we adopted elevated ALT as the diagnosis of suspected NAFLD, whereas the study by XM Zhang used ultrasonography [17]. The prevalence estimates from studies using ultrasound are generally lower than that from studies using ALT values [16].

The influence of race has long been in the discussion when accessing the difference of childhood NAFLD prevalence. Previous studies in America have revealed a higher prevalence of NAFLD in Hispanic and Mexican children [31,35]. Our study adopted the same ALT thresholds to diagnose the suspected NAFLD as the study in Chinese American children, but the Chinese children in our study demonstrated a higher prevalence of suspected NAFLD than the American Chinese children. Multiple factors may contribute to the comparatively high prevalence of suspected NAFLD in Chinese children, such as the difference of lifestyle. Furthermore, the blood test was only taken once in our study, whereas the elevated ALT was confirmed by repeating the test in the Chinese American study, which may decrease the prevalence of elevated ALT but increase the accuracy of the diagnosis [19].

Overweight and obesity, abdominal obesity, hyperuricemia, and elevated TC were all observed as correlates of suspected NAFLD in children. To date, this study is the most in-depth attention in exploring the correlates for suspected NAFLD in Chinese children. The relationship between obesity and NAFLD has been well established in previous studies [36]. The finding of hyperuricemia and TC as independent correlates of NAFLD is in line with the previous studies in both pediatric and adult NAFLD patients [37,38,39]. Our studies put additional evidence of the association in the general pediatric population, which may accelerate the adoption of hyperuricemia as a candidate diagnosis indicator for NAFLD in children. However, the population-based association data is scanty comparing with the clinical studies, further studies are still needed to verify the effects of potential risk factors of childhood NAFLD.

The major merits of our study are that the data came from a large representative sample of the Chinese population, the use of standardized protocols and data collection procedures, training of data collectors, as well as quality control assurance. In addition, all of the blood samples were analyzed in the same laboratory in the capital according to clinic laboratory standards, which can largely avoid measurement bias. However, there are also a number of limitations. First, the prevalence of suspected NAFLD may be overestimated because the biochemical test was only conducted once. Second, although the prevalence of hepatitis B virus (HBV) infection and hepatitis C virus (HCV) infection in Chinese children is low [40,41], because of the lack of tests for the serum makers of hepatitis B virus (HBV) and hepatitis C virus (HCV) in our study, the prevalence of suspected NAFLD may still be relatively overestimated. However, the prevalence of suspected NAFLD may also be underestimated because it is based on ALT serum level alone, which is not adequate as a single marker for diagnosing NAFLD [42]. Third, the lack of imaging and liver biopsy data limited our ability to confirm the diagnosis of NAFLD, so only the prevalence of suspected NAFLD could be assessed. Fourth, because of the cross-sectional nature of the study, no causality relationship can be assessed. Fifth, we could not include all possible correlates (hepatitis B virus infection, hepatitis C virus infection or other causes of liver disease) for analysis because of the availability of data.

## 5. Conclusions

To conclude, our study revealed the prevalence of suspected NAFLD in Chinese children for the first time, and explored its correlates. Our findings indicate that childhood suspected NAFLD is associated with the increasing childhood morbidities. Further epidemiological studies are still needed to better understand prevalence of childhood NAFLD using imaging and liver biopsies, and large-scale programs should also be launched to screen for NAFLD in the pediatric population in China.

## Figures and Tables

**Table 1:** Basic characteristics of the children with respect to gender and residence.

Characteristic	Total (*n* = 831)	Boys (*n* = 456)	Girls (*n* = 375)	Urban (*n* = 347)	Rural (*n* = 466)
Age (years)	12.39 ± 3.05	12.34 ± 3.07	12.45 ± 3.02	12.72 ± 3.02	11.91 ± 2.84 ^$^
Weight (kg)	39.64 ± 13.13	40.29 ± 13.92	38.86 ± 12.08	42.45 ± 13.28	37.56 ± 12.63 ^$^
Height (cm)	147.30 ± 15.85	148.24 ± 17.05	146.19 ± 14.24	150.63 ± 15.69	144.84 ± 15.54 ^$^
BMI (kg/m^2^)	17.77 ± 3.33	17.80 ± 3.38	17.72 ± 3.26	18.24 ± 3.33	17.42 ± 3.29 ^$^
WC (cm)	63.26 ± 9.61	64.09 ± 10.15	62.27 ± 8.82 *	64.43 ± 9.14	62.41 ± 9.86 ^$^
SBP (mmHg)	100.04 ± 13.02	100.48 ± 13.41	99.51 ± 12.54	100.76 ± 11.93	99.51 ± 13.76
DBP (mmHg)	66.67 ± 9.50	66.81 ± 9.48	66.50 ± 9.53	67.19 ± 9.39	66.28 ± 9.57
Hb (g/L)	137.65 ± 16.54	140.45 ± 16.31	134.26 ± 16.20 *	137.07 ± 15.64	137.94 ± 16.77
UA (μmol/L)	310.14 ± 84.99	328.74 ± 89.5	287.51 ± 73.14 *	314.12 ± 87.03	307.2 ± 84.24
TC (mmol/L)	3.88 ± 0.70	3.81 ± 0.70	3.96 ± 0.69 *	3.93 ± 0.71	3.84 ± 0.69
HDL (mmol/L)	1.44 ± 0.53	1.42 ± 0.35	1.46 ± 0.70	1.48 ± 0.72	1.41 ± 0.34
LDL (mmol/L)	2.21 ± 0.88	2.18 ± 1.05	2.24 ± 0.62	2.23 ± 0.93	2.18 ± 0.85
TG (mmol/L)	1.01 ± 0.72	0.98 ± 0.74	1.04 ± 0.68	1.02 ± 0.76	0.99 ± 0.69
ALT (IU/L)	13.00 (10.00–17.00)	14.00 (11.00–18.00)	12.00 (9.00–15.00) *	12.00 (9.00–17.00)	13.00 (10.00–17.00) ^$^

Data are means ± SD (standard deviation); * significantly different from boys (*p* < 0.05); ^$^ significantly different from urban children (*p* < 0.05).

**Table 2:** Basic characteristics of children with and without suspected NAFLD.

Characteristic	Suspected NAFLD (*n* = 75)	Without Suspected NAFLD (*n* = 756)	*p*-Value
Age (years)	12.85 ± 3.01	12.34 ± 3.05	0.173
Gender			
Boys (%)	49 (10.75%)	407 (89.25%)	0.056
Girls (%)	26 (6.93%)	349 (93.07%)	
Residence			
Urban (%)	29 (8.36%)	318 (91.64%)	0.593
Rural (%)	44 (9.44%)	422 (90.56%)	
Weight (kg)	44.96 ± 14.96	39.12 ± 12.83	<0.001 *
Height (cm)	149.24 ± 15.27	147.11 ± 15.91	0.282
BMI (kg/m^2^)	19.74 ± 4.61	17.57 ± 3.11	<0.001 *
WC (cm)	69.93 ± 11.68	62.61 ± 9.13	<0.001 *
SBP (mmHg)	103.95 ± 12.50	99.66 ± 13.02	0.008 *
DBP (mmHg)	69.54 ± 8.76	66.39 ± 9.53	0.008 *
Hb (g/L)	141.15 ± 19.90	137.30 ± 16.14	0.109
UA (μmol/L)	347.12 ± 86.86	306.47 ± 83.98	<0.001 *
TC (mmol/L)	4.20 ± 1.01	3.85 ± 0.65	0.004 *
HDL (mmol/L)	1.32 ± 0.36	1.45 ± 0.55	0.039 *
LDL (mmol/L)	2.43 ± 0.82	2.18 ± 0.88	0.021 *
TG (mmol/L)	1.49 ± 1.23	0.96 ± 0.63	<0.001 *
ALT (IU/L)	32.00 (27.00–50.00)	12.00 (10.00–15.00)	<0.001 *

Data are means ± SD or *n* (%); * significantly different (*p* < 0.05).

**Table 3:** Logistic regression analysis of the factors associated with childhood suspected NAFLD.

Characteristic	Adjusted OR (95% CI)	*p*-Value
BMI category		
<85th percentile ^r^	1.00	
Overweight and obesity	2.02 (1.04–3.93)	0.038
Abdominal obesity		
No ^r^	1.00	
Yes	2.76 (1.35–5.66)	0.006
Hyperuricemia ^a^		
No ^r^	1.00	
Yes	2.31 (1.38–3.89)	0.002
Elevated TC		
No ^r^	1.00	
Yes	4.70 (2.04–10.79)	<0.001

^r^ reference category; ^a^ UA > 327 μmol/L; variables included in the adjusted model were age, gender, residence, BMI category, abdominal obesity, hypertension, anaemia, hyperuricemia, and lipid disorders (elevated TC, low HDL, elevated LDL, elevated TG, and dyslipidemia).

## Abbreviations

The following abbreviations are used in this manuscript:

NAFLD	nonalcoholic fatty liver disease
CHNS	China Health and Nutrition Surveys
WC	waist circumference
BP	blood pressure
ALT	serum alanine aminotransferase
SBP	systolic blood pressure
DBP	diastolic blood pressure
Hb	hemoglobin
UA	serum uric acid
TC	total cholesterol
HDL	high-density lipoprotein cholesterol
LDL	low-density lipoprotein cholesterol
TG	triglyceride
OR	odds ratio
CI	confidence interval
